# *Campylobacter jejuni* HS:23 and Guillain-Barré Syndrome, Bangladesh

**DOI:** 10.3201/eid1508.090120

**Published:** 2009-08

**Authors:** Zhahirul Islam, Alex van Belkum, Alison J. Cody, Helen Tabor, Bart C. Jacobs, Kaisar A. Talukder, Hubert P. Endtz

**Affiliations:** International Centre for Diarrhoeal Disease Research, Bangladesh, Dhaka, Bangladesh (Z. Islam, K.A. Talukder, H.P. Endtz); Erasmus MC University Medical Center, Rotterdam, the Netherlands (Z. Islam, A. van Belkum, H.P. Endtz, B.C. Jacobs); University of Oxford, Oxford, United Kingdom (A.J. Cody); Laboratory Centre for Disease Control, Winnipeg, Manitoba, Canada (H. Tabor)

**Keywords:** Guillain-Barré syndrome, Campylobacter jejuni, bacteria, multilocus sequence type, Bangladesh, letter

**To the Editor**: Guillain-Barré syndrome (GBS) is an acute peripheral neuropathy triggered by a preceding infectious illness. Gastroenteritis caused by *Campylobacter jejuni* is the most frequently reported antecedent event (*1*). In Japan, South Africa, China, and Mexico, *Campylobacter* strains with certain Penner heat-stable (HS) serotypes, including HS:19 and HS:41, are overrepresented among isolates from GBS case-patients, compared with isolates from enteritis case-patients (*2*,*3*). Several studies indicate that *C. jejuni* HS:19 and HS:41 have a clonal population structure and suggest that these serotypes might have unique virulence properties that are intricately linked to development of GBS (*4*). However, data from the United Kingdom and the Netherlands suggest that such virulence properties may not be restricted to specific HS serotypes because many other serotypes can be cultured from patients with GBS (*5*). We report a non-HS:19 and non-HS:41 *C. jejuni* serotype and sequence type (ST)–3219 that are overrepresented among isolates from GBS patients in Bangladesh.

We conducted a prospective case-control study of the serotype and genotype of *C. jejuni* associated with GBS in Bangladesh. Case-patients were 97 persons with GBS admitted to Dhaka Medical College Hospital, Bangabandhu Sheikh Mujib Medical University, and Dhaka Central Hospital during July 2006–June 2007. All fulfilled the diagnostic criteria for GBS of the National Institute of Neurological Disorders and Stroke of the US National Institutes of Health (Bethesda, MD, USA) (*6*). The control group comprised 97 patients with other neurologic diseases, matched with case-patients by sex, age, and date of admission to the hospital. A second control group comprised 97 healthy family members of case-patients. Up to 3 stool samples were cultured from each case-patient and control.

*Campylobacter* strains were presumptively identified with Gram stain, oxidase, and hippurate hydrolysis tests and confirmed with a *C. jejuni* species-specific PCR. Serotyping was performed at the National Laboratory for Enteric Pathogens, Canadian Science Centre for Human and Animal Health, Winnipeg, Manitoba, Canada. All strains were serotyped according to the HS serotyping schemes of Penner et al. (*7*). To determine the class of lipooligosaccharides (LOS) locus in each of the *C. jejuni* strains, genomic DNA was isolated by using the DNeasy tissue kit (QIAGEN, Venlo, the Netherlands). PCR analysis was performed with primer sets specific for classes A, B, C, D, and E (*8*).

We isolated *C. jejuni* from fecal samples of 10 case-patients. *Campylobacter* strains were not isolated from the control groups (p<0.001). Serotyping of the 10 GBS-related strains showed 4 different HS serotypes. *C. jejuni* HS:23 was found in 5 (50%) strains; HS:19, in 2 (20%); HS:55 and HS:21, in 1 strain each. One strain was untypeable according to the HS typing scheme. In a collection of clinical *C. jejuni* isolated during the same period from patients with enteritis, HS:23 was encountered in 9 (28%) of 32 patients. Serotypes previously associated with GBS were HS:1, HS:2, HS:4, HS:4/50, HS:5, HS:10, HS:13/65, HS:16, HS:19, HS:23, HS:35, HS:37, HS:41, HS:44, and HS:64 (*5**,**9*).

Nine (90%) of the *C. jejuni* isolates from the case-patients had the class A or class B LOS, which are highly associated with the presence of ganglioside-mimicking structures in LOS (*10*). Godschalk et al. found that 14 (82%) of 17 GBS-associated isolates possessed a class A/B/C locus (*8*). Parker et al. (*10*) found that all GBS-related strains and 64% of the other clinical and environmental isolates belonged to LOS class A/B/C loci. The expression of ganglioside-mimicking structures in *Campylobacter,* LOS is considered essential for the induction of autoantibodies that lead to GBS. Godschalk et al. (*8*) demonstrated that specific genes involved in *C. jejuni* LOS biosynthesis are crucial for the induction of antiganglioside antibodies that lead to GBS.

We performed multilocus sequence typing to examine the overall genomic variation among 10 GBS-related *C. jejuni* strains. We identified 6 different STs among the GBS-related *C. jejuni* strains (Table). However, ST-3219 has a new combination of alleles and was identified in 4 strains. Concordantly, the analysis demonstrated that *C. jejuni* isolates with serotype HS:23 were all ST-3219. Of particular interest, ST-985 (BD-67) shared 5 alleles (*aspA, uncA, glnA, glyA, pgm*) with ST-3219 (Table).

**Table:** Serotyping and multilocus sequence typing analysis of *Campylobacter jejuni* strains associated with Guillain-Barré syndrome, Bangladesh*

Strain	Year	Disease	LOS class	Penner type(s)†	ST	Allele, no.						
						*aspA*	*glnA*	*gltA*	*glyA*	*pgm*	*tkt*	*uncA*
BD-07	2006	GBS	A	HS:19	22	1	3	6	4	3	3	3
BD-10	2006	GBS/MFS	B	HS:23	3219	10	27	33	19	10	5	7
BD-22	2006	GBS	B	HS:23	3219	10	27	33	19	10	5	7
BD-27	2006	GBS	A	UT	587	1	2	42	4	90	25	8
BD-34	2006	GBS	B	HS:23	3219	10	27	33	19	10	5	7
BD-39	2006	GBS	A	HS:19	660	1	3	6	4	54	91	3
BD-67	2007	GBS/MFS	B	HS:23	985	10	27	89	19	10	132	7
BD-74	2007	GBS/MFS	B	HS:23	3219	10	27	33	19	10	5	7
BD-75	2007	GBS	A	HS:55	587	1	2	42	4	90	25	8
BD-94	2007	GBS	E	HS:21	2109	4	7	10	4	10	7	1

*LOS, lipooligosaccharides; ST, sequence type; GBS, Guillain-Barré syndrome; HS, heat stable; MFS, Miller-Fisher syndrome; UT, untypeable. †Penner HS serotypes.

Our findings of a *C. jejuni* HS:23 serotype and ST-3219 that is highly prevalent among GBS-related *C. jejuni* strains from Bangladesh are consistent with previous observations that specific LOS types and serotypes are overrepresented among GBS-related *C. jejuni* strains. These observations support the hypothesis that, although a great variety of *C. jejuni* serotypes can be isolated from GBS patients in some geographic areas, specific clonal serotypes and multilocus types are prevalent in GBS patients in other places. The association of GBS with *C. jejuni* LOS class A/B/C is the only consistent finding when universal collections of GBS-associated strains are considered.
